# Supplementary material for: Sleep-active neuron specification and sleep induction require FLP-11 neuropeptides to systemically induce sleep
Source: eLife. 2016 Mar 7;5:e12499. doi: 10.7554/eLife.12499 (PMC4805538; doi:10.7554/eLife.12499)
Supplement: Supplementary file 1. — Table 1A, Genes with altered expression in aptf-1(gk794) mutant in pretzel-stage embryos. Table 1B, Genes with altered expression in aptf-1(gk794) mutant during L4 larvae sleep. DOI: http://dx.doi.org/10.7554/eLife.12499.015 [file elife-12499-supp1.docx]

**Supplementary File 1**

**Table 1A,** Genes with altered expression in *aptf-1(gk794)* mutant in pretzel-stage embryos.

| **AgilentID** | **Genbank**  **Accession** | **Gene**  **Symbol** | **log2FC**  ***aptf-1* - N2** | **P.Value**  ***aptf-1* - N2** | **FDR**  ***aptf-1* - N2** |
| --- | --- | --- | --- | --- | --- |
| A_12_P112897 | NM_062899 | *aptf-1* | -2.04 | 2.50E-07 | 0.44% |
| A_12_P119509 | NM_077540 | *sto-3* | -1.96 | 8.36E-06 | 2.16% |
| A_12_P104350 | NM_073426 | H19N07.3 | -1.77 | 1.65E-06 | 0.84% |
| A_12_P118033 | NM_001083192 | C10C6.7 | -1.76 | 1.23E-06 | 0.84% |
| A_12_P150622 | NM_001029581 | *flp-11* | -1.74 | 1.64E-06 | 0.84% |
| A_12_P114872 | NM_001029581 | *flp-11* | -1.73 | 2.45E-06 | 0.97% |
| A_12_P150620 | NM_001029581 | *flp-11* | -1.72 | 8.52E-06 | 2.16% |
| A_12_P157510 | NM_001083192 | C10C6.7 | -1.71 | 4.00E-08 | 0.14% |
| A_12_P150623 | NM_001029581 | *flp-11* | -1.70 | 2.15E-05 | 4.01% |
| A_12_P151791 | NM_001083191 | C10C6.7 | -1.69 | 2.15E-06 | 0.95% |
| A_12_P150621 | NM_001029581 | *flp-11* | -1.52 | 1.20E-06 | 0.84% |
| A_12_P110452 | NM_001029582 | *flp-11* | -1.48 | 5.64E-06 | 1.76% |
| A_12_P158070 | NM_075064 | *fbxa-192* | -1.41 | 1.67E-05 | 3.29% |
| A_12_P112651 | NM_001029583 | *flp-11* | -1.37 | 1.11E-06 | 0.84% |
| A_12_P157880 | NM_075064 | *fbxa-192* | -1.29 | 5.96E-06 | 1.76% |
| A_12_P132316 | NC_003283 | F57G4.7 | -1.29 | 1.41E-05 | 2.95% |
| A_12_P130231 | NM_001029581 | *flp-11* | -1.25 | 1.05E-05 | 2.32% |
| A_12_P132315 | NC_003283 | F57G4.7 | -1.17 | 1.03E-05 | 2.32% |
| A_12_P105169 | NM_074761 | *str-15* | 1.57 | 3.39E-06 | 1.20% |

**Table 1B,** Genes with altered expression in *aptf-1(gk794)* mutant during L4 larvae sleep.

| **AgilentID** | **Genbank**  **Accession** | **Gene**  **Symbol** | **log2FC**  ***aptf-1* - N2** | **P.Value**  ***aptf-1* - N2** | **FDR**  ***aptf-1* - N2** |
| --- | --- | --- | --- | --- | --- |
| A_12_P135877 | NM_061886 | *bath-13* | -1.76 | 5.56E-06 | 2.64% |
| A_12_P104350 | NM_073426 | H19N07.3 | -1.71 | 2.26E-05 | 4.96% |
| A_12_P110452 | NM_001029582 | *flp-11* | -1.37 | 1.68E-06 | 1.40% |
| A_12_P130231 | NM_001029582 | *flp-11* | -1.30 | 1.32E-06 | 1.40% |
| A_12_P150623 | NM_001029581 | *flp-11* | -1.30 | 1.03E-05 | 3.34% |
| A_12_P114872 | NM_001029581 | *flp-11* | -1.29 | 6.32E-06 | 2.64% |
| A_12_P102704 | NM_060180 | *rnh-1.3* | -1.27 | 2.54E-05 | 4.96% |
| A_12_P150620 | NM_001029581 | *flp-11* | -1.25 | 7.57E-07 | 1.26% |
| A_12_P112651 | NM_001029583 | *flp-11* | -1.24 | 2.54E-07 | 0.85% |
| A_12_P150622 | NM_001029581 | *flp-11* | -1.00 | 1.70E-05 | 4.50% |
| A_12_P176209 | BJ803681 | C06C3.11 | -0.88 | 4.62E-06 | 2.64% |
| A_12_P113421 | NM_063953 | R03D7.2 | -0.81 | 2.68E-05 | 4.96% |
| A_12_P114497 | NM_001028353 | Y57G11C.40 | -0.76 | 2.03E-05 | 4.84% |
| A_12_P102123 | NM_077668 | *zig-2* | -0.68 | 1.75E-05 | 4.50% |
| A_12_P181070 | NM_001029486 | *nspc-12* | 1.17 | 5.82E-06 | 2.64% |
| A_12_P100141 | NM_001026194 | *srh-2* | 1.44 | 2.61E-05 | 4.96% |
| A_12_P155805 | NM_001083208 | F13E9.15 | 1.55 | 1.10E-05 | 3.34% |
| A_12_P115454 | NM_061482 | F45D11.14 | 4.22 | 1.07E-05 | 3.34% |
