# Supplementary material for: Sleep-active neuron specification and sleep induction require FLP-11 neuropeptides to systemically induce sleep
Source: eLife. 2016 Mar 7;5:e12499. doi: 10.7554/eLife.12499 (PMC4805538; doi:10.7554/eLife.12499)
Supplement: Supplementary file 2. — A list of C. elegans strains that were used for this study. DOI: http://dx.doi.org/10.7554/eLife.12499.016 [file elife-12499-supp2.docx]

**Supplementary File 2**

***C. elegans* strains used**

**AQ2967:** *IjIs133[unc-47::GCaMP3-SL2-tagRFP-T].* (Gift from Victoria Butler and Bill Schafer)

**AX1792:** *dbEx721[npr-4::mCherry + unc-122p::GFP].* (Gift from Mario de Bono)

**BZ555:** *egIs1[dat-1p::GFP].*

**CZ521:** *juIs8[pSC382(unc-25::GFP)].* (Gift from Yishi Jin)

**EG1285:** *oxIs12[unc-47p::GFP + lin-15(+)]X.*

**HBR227:** *aptf-1(gk794)II.* (10xbc)

**HBR499***: aptf-1(gk794) II; ynIs40[flp-11p::GFP] V.*

**HBR507:** *flp-11(tm2706)X.* (7xbc)

**HBR546:** *goeIs102[paptf-1::ChR2::mKate2-aptf-1 3'UTR, unc-119(+)].* (2xbc)

**HBR739:** *unc-119(ed3)III, goeEx290[WRM0622A_F09(pRedFlp-Hgr)(C10C6.7[20642]*

*::S0001_pR6K_Amp_2xTY1ce_EGFP_FRT_rpsl_neo_FRT_3xFlag)dFRT::unc-119-Nat].*

**HBR762:** *C10C6.7(goe3)IV.*

**HBR764:** *C10C6.7(goe5)IV.*

**HBR777:** *unc-119(ed3)III, goeEx314[psto-3::d1mgfp::unc-54 3'UTR, unc-119(+)].*

**HBR778:** *aptf-1(gk794) II, goeEx290[(WRM0622A_F09(pRedFlpHgr)(C10C6.7[20642]*

*::S0001_pR6K_Amp_2xTY1ce_EGFP_FRT_rpsl_neo_FRT_3xFlag)dFRT::unc-119-Nat)].*

**HBR843:** *aptf-1(gk794)II, unc-119(ed3)III, goeEx338[paptf-1::tfap2β::mKate2-aptf-1 3’UTR, punc-122::GFP, unc-119(+)].*

**HBR868:** *goeIs118[paptf-1::SL1-GCaMP3.35-SL2::mKate2-aptf-1 3'UTR, unc-119(+)].* (2xbc)

**HBR887:** *unc-119(ed3)III, goeEx355[pnmr-1::d1mgfp::unc-54 3'UTR, unc-119(+)].*

**HBR893:** *lim-6(nr2073)X, goeIs118[paptf-1::SL1-GCaMP3.35-SL2::mKate2-aptf-1 3'UTR, unc-119(+)].*

**HBR914:** *lim-6(tm4836)X.* (7xbc)

**HBR935:** *flp-11(tm2706)X, goeIs102[paptf-1::ChR2::mKate2-aptf-1 3'UTR, unc-119(+)].*

**HBR973:** *lim-6(tm4836)X, IjIs133[unc-47::GCaMP3-SL2-tagRFP-T].*

**HBR1009:** *flp-11(tm2706)X, goeIs118[paptf-1::SL1-GCaMP3.35-SL2::mKate2-aptf-1 3'UTR, unc-119(+)].*

**HBR1021:** *goeIs240[phsp-16.2::flp-11::SL2-mKate2-unc-54 3’UTR, unc-119(+)].* (2xbc)

**HBR1026:** *npr-22(ok1598)IV, goeIs240[phsp-16.2::flp-11::SL2-mKate2-unc-54 3’UTR, unc-119(+)].*

**HBR1068:** *frpr-3(ok3302)V, goeIs240[phsp-16.2::flp-11::SL2-mKate2-unc-54 3’UTR, unc-119(+)].*

**HBR1080:** *npr-22(ok1598)IV, frpr-3(ok3302)V, npr-4(tm1782)X, goeIs240[phsp-16.2::flp-11::SL2-mKate2-unc-54 3’UTR, unc-119(+)].*

**HBR1081:** *npr-22(ok1598)IV, frpr-3(ok3302)V, goeIs240[phsp-16.2::flp-11::SL2-mKate2-unc-54 3’UTR, unc-119(+)].*

**HBR1082:** *frpr-3(ok3302)V, npr-4(tm1782)X, goeIs240[phsp-16.2::flp-11::SL2-mKate2-unc-54 3’UTR, unc-119(+)].*

**HBR1083:** *npr-22(ok1598)IV, npr-4(tm1782)X, goeIs240[phsp-16.2::flp-11::SL2-mKate2-unc-54 3’UTR, unc-119(+)].*

**HBR1084:** *npr-4(tm1782)X, goeIs240[phsp-16.2::flp-11::SL2-mKate2-unc-54 3’UTR, unc-119(+)].*

**HBR1085:** *npr-22(ok1598)IV, frpr-3(ok3302)V, npr-4(tm1782)X.* (3xbc)

**HBR1099:** *unc-119(ed3)III, goeEx386[WRM0635D_B04(pRedFlp-Hgr)*

*(H19N07.3[21364]::S0001_pR6K_Amp_2xTY1ce_EGFP_FRT_rpsl_neo_FRT_3xFlag)dFRT::unc-119-Nat].*

**HBR1100:** *aptf-1(gk794)II, unc-119(ed3)III, goeEx386[WRM0635D_B04(pRedFlp-Hgr)*

*(H19N07.3[21364]::S0001_pR6K_Amp_2xTY1ce_EGFP_FRT_rpsl_neo_FRT_3xFlag)dFRT::unc-119-Nat ].*

**HBR1205:** *flp-11(tm2706)X, goeEx457[pflp-11::flp-11-SL2::mKate2-unc-54 3'UTR, unc-119(+)].*

**HBR1206:** *aptf-1(gk794)II, unc-119(ed3)III, goeEx458[paptf-1::flp-11-SL2::mKate2-unc-54 3'UTR, unc-119(+)].*

**HBR1209:** *goeIs290[pfrpr-3::mKate2::unc-54 3'UTR, unc-119(+)].* (2xbc)

**HBR1210:** *goeIs290[pfrpr-3::mKate2::unc-54 3'UTR, unc-119(+)], bwIs2[flp-1::GFP + (pRF4)rol-6(su1006)].*

**HBR1211:** *goeIs290[pfrpr-3::mKate2::unc-54 3'UTR, unc-119(+)], oxIs12 [unc-47p::GFP + lin-15(+)].*

**HBR1212:** *goeIs290[pfrpr-3::mKate2:: unc-54 3'UTR, unc-119(+)], egIs1[dat-1p::GFP].*

**HBR1213:** *otEx2411[gcy-13(prom1)::GFP + unc-122].*

**HBR1214:** *goeIs290[pfrpr-3::mKate2:: unc-54 3'UTR, unc-119(+)], otEx2411[gcy-13(prom1)::GFP + unc-122::GFP].*

**HBR1215:** *goeIs290[pfrpr-3::mKate2::unc-54 3'UTR, unc-119(+)], otIs138[ser-2prom3::GFP + rol-6]X.*

**HBR1216:** *goeIs290[pfrpr-3::mKate2::unc-54 3'UTR, unc-119(+)], goeEx355[pnmr-1::d1mgfp::unc-54 3'UTR, unc-119(+)].*

**HBR1217:** *goeIs290[pfrpr-3::mKate2::unc-54 3'UTR, unc-119(+)], goeEx314[psto-3::d1mgfp::unc-54 3'UTR, unc-119(+)].*

**HBR1261:** *goeIs288[pflp-11::mKate2::unc-54 3'UTR, unc-119(+)].* (2xbc)

**HBR1290:** *npr-22(ok1598)IV.* (4xbc)

**HBR1291:** *npr-4(tm1782)X.* (4xbc)

**HBR1292:** *frpr-3(ok3302)V.* (4xbc)

**HBR1304:** *goeIs290[pfrpr-3::mKate2::unc-54 3'UTR, unc-119(+)], zfIs6[lgc-55::GFP].*

**HBR1318:** *unc-119(ed3)III, goeIs297[WRM0629B_F01(pRedFlp-Hgr)(C26F1.6[27788]::S0001_pR6K_Amp_2xTY1ce_EGFP_FRT_rpsl_neo_FRT_3xFlag)dFRT::unc-119-Nat); unc-119(+)].*

**HBR1330:** *goeIs285[pnpr-22::mKate2::unc-54 3'UTR, unc-119(+)].* (2xbc)

**HBR1331:** *goeIs285[pnpr-22::mKate2::unc-54 3'UTR, unc-119(+)], bwIs2[flp-1::GFP + (pRF4)rol-6(su1006)].*

**HBR1332:** *goeIs285[pnpr-22::mKate2::unc-54 3'UTR, unc-119(+)], oxIs12[unc-47p::GFP + lin-15(+)].*

**HBR1333:** *goeIs285[pnpr-22::mKate2::unc-54 3'UTR, unc-119(+)], egIs1[dat-1p::GFP].*

**HBR1334:** *goeIs285[pnpr-22::mKate2::unc-54 3'UTR, unc-119(+)], otEx2411[gcy-13(prom1)::GFP + unc-122.*

**HBR1335:** *goeIs285[pnpr-22::mKate2::unc-54 3'UTR, unc-119(+)], otIs138[ser-2prom3 + rol-6]X.*

**HBR1336:** *goeIs285[pnpr-22::mKate2::unc-54 3'UTR, unc-119(+)], goeEx355[pnmr-1::d1mgfp::unc-54 3'UTR, unc-119(+)]].*

**HBR1337:** *goeIs285[pnpr-22::mKate2::unc-54 3'UTR, unc-119(+)], goeEx314[psto-3::d1mgfp::unc-54 3'UTR, unc-119(+)].*

**HBR1338:** *goeIs285[pnpr-22::mKate2::unc-54 3'UTR, unc-119(+)], zfIs6[lgc-55::GFP].*

**HBR1355:** *unc-119(ed3)III, goeEx517[unc-119(ed3) III; WRM0616D_E08(pRedFlp-Hgr)(C16D6.2[36626]::S0001_pR6K_Amp_2xTY1ce_EGFP_FRT_rpsl_neo_FRT_3xFlag)dFRT::unc-119-Nat].*

**HBR1356:** *unc-119(ed3)III, goeIs305[unc-119(ed3) III; WRM0620B_D12(pRedFlp-Hgr)(Y59H11AL.1[36588]::S0001_pR6K_Amp_2xTY1ce_EGFP_FRT_rpsl_neo_FRT_3xFlag)dFRT::unc-119-Nat].*

**HBR1359:** *aptf-1(gk794)II, juIs8[pSC382(unc-25::GFP)]*.

**HBR1360:** *aptf-1(gk794)II, goeIs288[pflp-11::mKate2::unc-54 3'UTR, unc-119(+)].*

**HBR1364:** *goeIs303[pflp-11::rpl-1a-GFP::flp-11 3'UTR, unc-119(+)].* (2xbc) (a gift from Jan Konietzka)

**HBR1407:** *aptf-1(gk794)II*, *oxIs12[unc-47p::GFP + lin-15(+)]X*.

**HBR1415:** *goeEx538[phsp16.2::flp-10::SL2-mKate2-unc-54 3’UTR, unc-119(+)]].*

**HBR1416:** *goeEx539[phsp16.2::flp-20::SL2-mKate2-unc-54 3’UTR, unc-119(+)]].*

**HBR1443:** *aptf-1(gk794)II, goeIs303[pflp-11::rpl-1a-GFP::flp-11 3'UTR, unc-119(+)].*

**HBR1444:** *aptf-1(gk794)II, goeIs303[pflp-11::rpl-1a-GFP::flp-11 3'UTR, unc-119(+)], goeEx338[paptf-1::tfap2β::mKate2-aptf-1 3’UTR, punc-122::GFP, unc-119(+)].*

**MU1085:** *bwIs2[flp-1::GFP + (pRF4)rol-6(su1006)].*[[80](#_ENREF_80)]

**N2:** wild type

**NY2009:** *lin-15(n765)ts, ynIs9[phsp16.2::flp-1, lin-15B].* (Gift from Chris Li)

**NY2040:** *ynIs40[flp-11p::GFP] V.*

**OH110:** *lim-6(nr2073)X.*

**OH1422:** *otIs138[ser-2prom3 + rol-6]X.*

**TM1488:** *sto-3(tm1488)X.*

**QW122:** *zfIs6[Is lgc-55::GFP (2.1)].* (Gift from Mark Alkema)
