# Supplementary material for: Sleep-active neuron specification and sleep induction require FLP-11 neuropeptides to systemically induce sleep
Source: eLife. 2016 Mar 7;5:e12499. doi: 10.7554/eLife.12499 (PMC4805538; doi:10.7554/eLife.12499)
Supplement: Supplementary file 3. — A list of primers that were used for this study. DOI: http://dx.doi.org/10.7554/eLife.12499.017 [file elife-12499-supp3.docx]

**Supplementary File 3**

**Primers used**

*flp-11(tm2706)*

CAG GAG TTG TTC GAG CAG AA

TCG TCC AAT GGA GAC CTC TT

TAG CCG CTC GTC TCA CTT TT

*frpr-3(ok3302)*

AAG GAA TCG CGC TCT CAT AA

GTT TGT CCT CGC GGT AGG TA

GTG GCA AGT GTG AAA CGA GA

*lim-6(tm4836)*

AAC CCT CTC TCG GAA CAG TG

GGC TGA ACA AGA AAT CCG CA

AGA CAC CCA CAA CCA GAA CA

*npr-4(tm1782)*

AGC TGC AAA ACC AAC AAA CG

TGA CCA AAT CCA GCT GAA GA

CGG TTG GAG TGA TTG CTG TA

*npr-22(ok1598)*

ACG ACG ACA GCC TCT GAA AT

AAG TAC ATG GCA CGT TAA AAT CAA

TGG CAA AAT TTA GTT CGC AGT
