# Supplementary material for: Sleep-active neuron specification and sleep induction require FLP-11 neuropeptides to systemically induce sleep
Source: eLife. 2016 Mar 7;5:e12499. doi: 10.7554/eLife.12499 (PMC4805538; doi:10.7554/eLife.12499)
Supplement: Supplementary file 4. — A list of DNA constructs (Plasmids and Fosmids) that were used for this study. DOI: http://dx.doi.org/10.7554/eLife.12499.018 [file elife-12499-supp4.docx]

**Supplementary File 4**

**DNA constructs that were used**

**Gateway entry clones:**

*paptf-1*: as described in Turek et al. 2013

*pflp-11*: 2,746 bp directly upstream of *flp-11* coding region

*phsp-16.2*: pCM1.56 as described in Jones et al. 1986

*pfrpr-3*: 2,029 bp directly upstream of *frpr-3* coding region

*pnpr-4*: 4,000 bp directly upstream of *npr-4* isoforms c and d

*pnpr-22*: 2,059 bp directly upstream of *npr-22* coding region

*psto-3*: as described in Turek et al. 2013

*pnmr-1*: pIR11 as described in Turek et al. 2013

*SL1-GCaMP3.35-SL2*: as described in Schwarz et al. 2011

*mKate2*: as described in Schwarz et al. 2011

*tfap2β*: TFAP2β mouse; codon-optimized for *C. elegans* as described in Redemann 2001

*flp-11*: *flp-11* isoform b coding region

*flp-10: flp-10* coding region including a synthetic intron of 51 bp length replacing the original intron

*flp-20: flp-20* coding region

*rpl-1a-GFP*: N-terminus GFP-tagged *rpl-1a* with 3 synthetic introns; codon-optimized for *C. elegans*

*Channelrhodopsin-2*: pIR60 as described in Turek et al. 2013

*d1mgfp*: as described in Turek et al. 2013

*unc-54 3’UTR*: as described in Schwarz et al. 2011

*flp-11 3’UTR*: 356 bp directly downstream of *flp-11* isoform b

*SL2-mKate2-unc-54 3’UTR*: as described in Turek et al. 2013

*mKate2-unc-54 3’UTR*: as described in Schwarz et al. 2011

*mKate2-aptf-1 3’UTR*: as described in Turek et al. 2013

**Gateway final constructs:**

*paptf-1::SL1-GCaMP3.35-SL2::mKate2-aptf-1 3’UTR*

*pflp-11::mKate2::unc-54 3’UTR*

*pflp-11::rpl-1a-GFP::flp-11 3'UTR*

*paptf-1::tfap2β::mKate2-aptf-1 3’UTR*

*pflp-11::flp-11::SL2-mKate2-unc-54 3’UTR*

*paptf-1::flp-11::SL2-mKate2-unc-54 3’UTR*

*paptf-1::Channelrhodopsin-2::mKate2-aptf-1 3’UTR*

*phsp-16.2::flp-11::SL2-mKate2-unc-54 3’UTR*

*pfrpr-3::mKate2::unc-54 3’UTR*

*pnpr-22::mKate2::unc-54 3’UTR*

*psto-3::d1mgfp::unc-54 3’UTR*

*pnmr-1::d1mgfp::unc-54 3’UTR*

*phsp16.2::flp-10::SL2-mKate2-unc-54 3’UTR*

*phsp16.2::flp-20::SL2-mKate2-unc-54 3’UTR*

**Fosmids**

***gfp*-tagged *frpr-3* fosmid:** *WRM0629B_F01(pRedFlp-Hgr)(C26F1.6[27788]::S0001_pR6K_Amp_2xTY1ce_EGFP_FRT_rpsl_neo_FRT_3xFlag)dFRT::unc-119-Nat*

***gfp*-tagged *npr-4* fosmid:** *WRM0616D_E08(pRedFlp-Hgr)(C16D6.2[36626]::S0001_pR6K_Amp_2xTY1ce_EGFP_FRT_rpsl_neo_FRT_3xFlag)dFRT::unc-119-Nat*

***gfp*-tagged *npr-22* fosmid:** *WRM0620B_D12(pRedFlp-Hgr)(Y59H11AL.1[36588]::S0001_pR6K_Amp_2xTY1ce_EGFP_FRT_rpsl_neo_FRT_3xFlag)dFRT::unc-119-Nat*

***gfp*-tagged C10C6.7 fosmid:** *WRM0622A_F09(pRedFlp-Hgr)(C10C6.7[20642]::S0001_pR6K_Amp_2xTY1ce_EGFP_FRT_rpsl_neo_FRT_3xFlag)dFRT::unc-119-Nat*

***gfp*-tagged H19N07.3 fosmid:** *WRM0635D_B04(pRedFlp-Hgr)(H19N07.3[21364]::S0001_pR6K_Amp_2xTY1ce_EGFP_FRT_rpsl_neo_FRT_3xFlag)dFRT::unc-119-Nat*
